# Kinetics and Predicted Structure of a Novel Xylose Reductase from *Chaetomium thermophilum*

**DOI:** 10.3390/ijms20010185

**Published:** 2019-01-06

**Authors:** Julian Quehenberger, Tom Reichenbach, Niklas Baumann, Lukas Rettenbacher, Christina Divne, Oliver Spadiut

**Affiliations:** 1 Research Division Biochemical Engineering, Institute of Chemical, Environmental and Bioscience Engineering, Faculty of Technical Chemistry, TU Wien, 1060 Vienna, Austria; julian.quehenberger@tuwien.ac.at (J.Q.); bau.nik@hotmail.com (N.B.); lukasalexanderrettenbacher@gmail.com (L.R.); 2 KTH School of Engineering Sciences in Chemistry, Biotechnology and Health, SE-100 44 Stockholm, Sweden; tomre@kth.se (T.R.); divne@kth.se (C.D.)

**Keywords:** xylose reductase, *Chaetomium thermophilum*, kinetics, structure, homology model, cofactor binding, stability

## Abstract

While in search of an enzyme for the conversion of xylose to xylitol at elevated temperatures, a xylose reductase (XR) gene was identified in the genome of the thermophilic fungus *Chaetomium thermophilum*. The gene was heterologously expressed in *Escherichia coli* as a His6-tagged fusion protein and characterized for function and structure. The enzyme exhibits dual cofactor specificity for NADPH and NADH and prefers D-xylose over other pentoses and investigated hexoses. A homology model based on a XR from *Candida tenuis* was generated and the architecture of the cofactor binding site was investigated in detail. Despite the outstanding thermophilicity of its host the enzyme is, however, not thermostable.

## 1. Introduction

*Chaetomium thermophilum* is a thermophilic fungus of the phylum Ascomycota. It is one of the most thermophile Eukaryotes, growing optimally at temperatures of 50–55 °C on rotting organic matter [1]. The organism is a mycelium-forming fungus with multinucleate hyphae, perforated by septa—similar to other filamentous ascomycetes, such as *Neurospora crassa* or *Aspergillus* sp. The complete nuclear and mitochondrial genomes of *C. thermophilum* are sequenced and the nuclear genome is estimated to be 28.3 Mbp with 7227 open reading frames while the mitochondrial genome is 127 kbp with 15 protein coding genes [2].

Due to the host’s outstanding thermostability, it is a promising source of stable enzymes. In total, 191 Protein Data Bank (PDB) entries for *C. thermophilum* have been deposited to this date; a number that has been rapidly increasing in recent years compared to only 19 entries until the year 2013 [1]. From an industrial point of view the organism is especially interesting for prospecting thermostable enzymes that are exclusively present in Eukaryotes. These enzymes cannot be found in extreme thermophiles (growth at ≥85 °C), a group of organisms that are only found in the prokaryotic Domains Bacteria and Archaea [3].

The enzyme D-xylose reductase (EC 1.1.1.307) belongs to the aldose reductase family. It is commonly found in filamentous fungi and yeasts, and catalyzes the reduction of d-xylose to the sugar alcohol xylitol. This reaction marks the initial step of the fungal xylose catabolism, eventually leading to the pentose phosphate pathway via oxidation of xylitol to d-xylulose by the enzyme xylitol dehydrogenase. Generally, wild type XRs strongly favor NADPH as cofactor, while dual cofactor specificity for NADH and NADPH was only observed in some cases [4,5,6,7]. Only a single XR was reported that preferred NADH over NADPH [8]. More recently, several XRs have been generated via mutation with increased affinity towards NADH in order to circumvent the problem of cofactor balance in enzyme cascades with NAD^+^-dependent xylitol dehydrogenases [9,10,11,12,13,14,15]. The core structure of XRs is a (β/α)_8_-barrel fold composed of eight alpha-helices running antiparallel to eight beta sheets in the center of the barrel [16]. Commonly, XRs are found to form dimers in their native state [17].

XRs are of significant industrial interest due to their importance for the utilization of hemicellulosic biomass as an energy and carbon source for ethanol production in yeasts [18,19], as well as for use as a biocatalyst for the conversion of xylose to the value added product xylitol. Xylitol is a naturally occurring, low-caloric sugar alcohol with a relative sweetening power comparable with sucrose [20]. Due to its anticariogenic properties and low glycemic index, it is widely used in chewing gum, food and beverages as well as in the pharmaceutical industry [21,22]. In a recent estimation, the global market for xylitol was expected to reach USD 1.37 billion by 2025 with an annual growth rate of 6.6% [23].

A considerable number of different XRs has been investigated with regard to stability and reactivity and mutated variants have been produced with the aim of increased activity and shift in cofactor preference, with examples in references [9,10,11,13,15]. Apart from the general desire to improve the stability of industrially relevant enzymes, a more specific benefit of thermostable XRs could be the possibility of direct utilization of heat-treated hemicellulosic biomass for the conversion of liberated xylose to xylitol. Nevertheless, most of the described XRs are mesophilic, whereas the highest described temperature optimum is 45–55 °C for an XR from *N. crassa* [24], and 50 °C for an XR from *Candida tenuis* [6].

In order to find an XR with high activity at elevated temperatures, we chose to investigate the genome of one of the most thermostable Eukaryotes, namely *C. thermophilum*, with the aim to heterologously express, purify and characterize a novel XR.

## 2. Results and Discussion

### 2.1. Protein Production

After 34 h total fermentation time, 230 g/L wet cell weight (corresponding to 62 g/L dry cell weight (DCW)) were harvested. Crude extract derived from 230 g wet cell weight contained a total activity of 360 U, resulting in a titer of 360 U/L or 5.8 U/g DCW. The space time yield (volumetric productivity) equals 10.6 U/L/h.

### 2.2. Protein Purification

**Figure 1:**
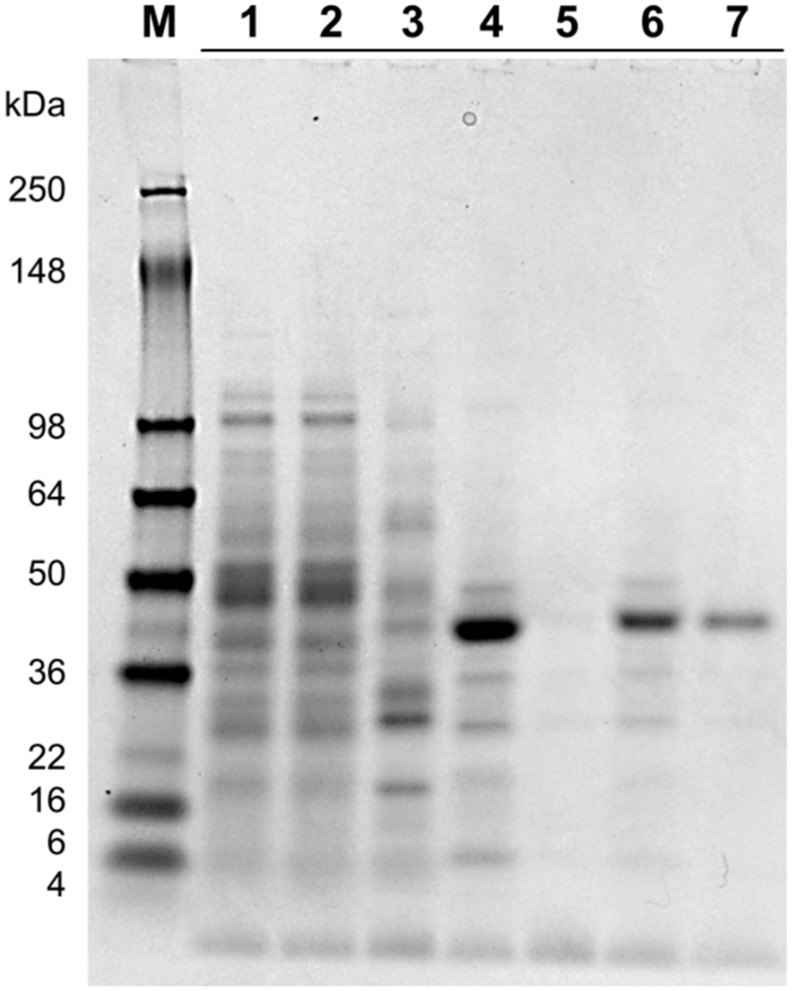
SDS-PAGE image to evaluate the purification process of *Ct*XR. The target protein is located at a height between 36 and 50 kDa with an estimated mass of 41 kDa. M: Protein ladder (SeeBlue Plus2 prestained standard); 1: Crude extract (diluted 1:10); 2: IMAC Flow through (1:10); 3: IMAC Eluate 1 (pre elution peak; 1:1); 4: IMAC Elution peak (determined by a jump in the UV absorption signal in the eluate; 1:10); 5: IMAC Eluate 2 (post elution peak; 1:5); 6: Spin filter Flow through (1:40); 7: SEC Peak (collected fraction based on a jump in the UV absorption signal; 1:20).

Recombinant His6-tagged *Ct*XR was purified to near homogeneity and the apparent molecular mass (Mw), estimated with SDS-PAGE to be approximately 41 kDa, agreed with the theoretical Mw of 39,244 Da. As shown in Table 1, loss of active *Ct*XR during purification was significant. Mass overload during immobilized metal affinity chromatography (IMAC) caused product loss in the flow through. Poor product retention during spin filtration and cutting of product peak during size exclusion chromatography (SEC), to increase purity, led to further product loss. This results in a total recovery of only 3% but a high purification factor of 25. Product size and purity of the various fractions can be seen in the SDS-PAGE image in Figure 1.

### 2.3. Biochemical Characterization

#### 2.3.1. Enzyme Activity

**Figure 2:**
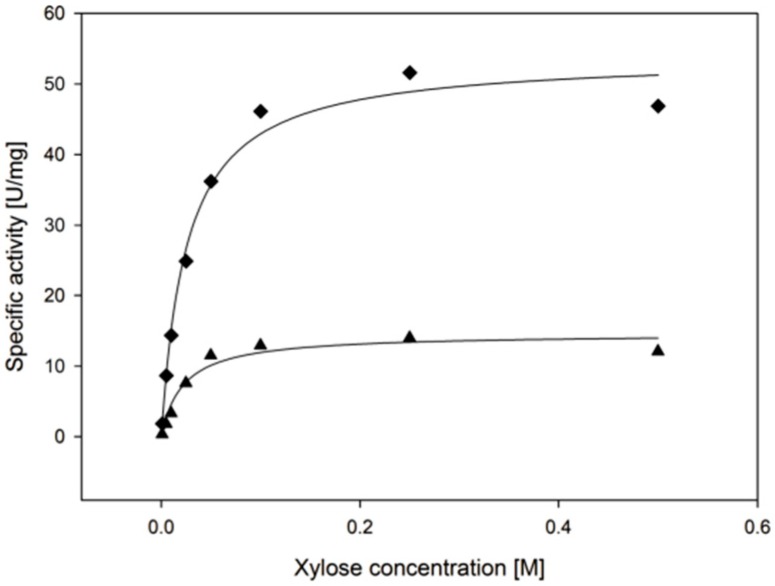
Michaelis-Menten curves at ▲ 30 °C and ◆ 55 °C. The concentration of d-xylose was varied and concentration of NADPH was kept at 0.4 mM.

*Ct*XR was kinetically characterized at 30 and 55 °C with the substrate D-xylose in limiting concentrations while NADPH was provided in non-limiting concentrations (Figure 2). k_cat_ increases from 30 to 55 °C while there is only little change in K_M_. (Table 2). This means, that while the turnover rate (k_cat_) increases, the apparent affinity towards the substrate only decreases slightly with rising temperature.

#### 2.3.2. pH Optimum

**Figure 3:**
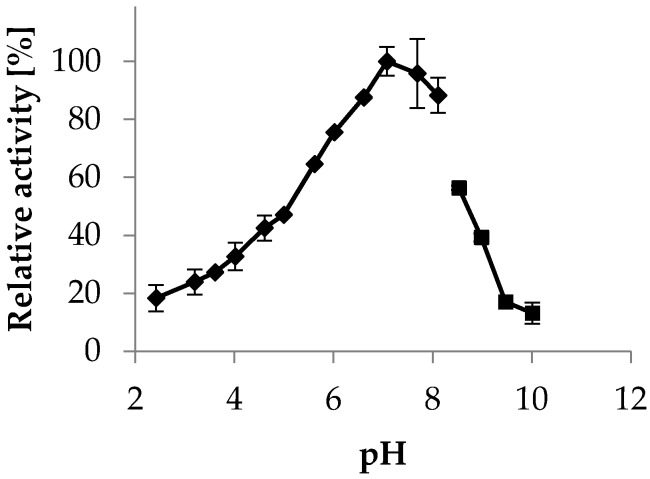
Xylose reductase activity at different pH values. Measurements were taken at 30 °C in a citric acid-disodium phosphate buffer (McIlvaine buffer) [25] (◆ pH 2.4–8.1) and 50 mM glycine buffer (■ pH 8.5–10.0).

The pH optimum of most xylose reductases described in the literature is at pH 6.5. We determined a pH optimum of *Ct*XR in a citrate-disodium phosphate buffer at pH 7.0 (Figure 3). In 50 mM citrate and 50 mM phosphate buffer the pH optimum was found at pH 6.0 and 6.5, respectively (Supplementary Figure S1). These results indicate that increased ionic strength, or, more specifically, a higher phosphate concentration, leads to an apparent shift of the pH optimum towards higher values, as the citrate disodium phosphate buffer contains 165 mM phosphate at pH 7.

#### 2.3.3. Cofactor Preference

**Figure 4:**
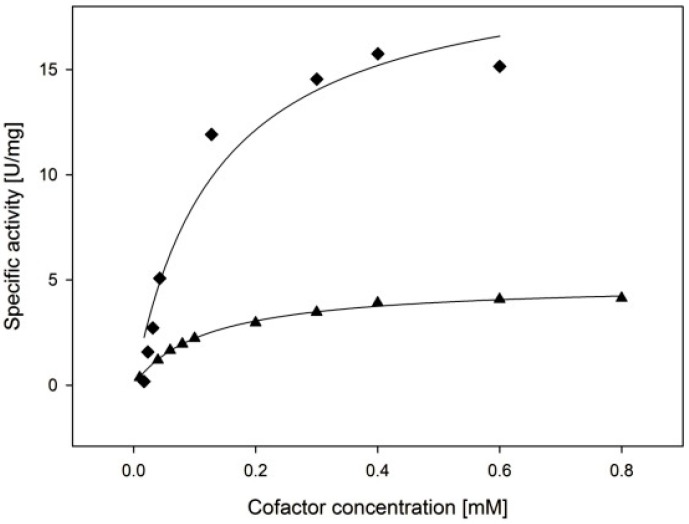
Michaelis-Menten curves for determination of cofactor preference: ◆ 0.5 M d-xylose, variation of NADPH; ▲ 0.5 M d-xylose, variation of NADH. Measurements were taken at 30 °C.

To test whether *Ct*XR preferred NADPH or NADH as cofactor, K_M_, k_cat_ and the catalytic efficiency were determined (Figure 4). The enzyme shows dual cofactor specificity and similar K_M_ values for NADPH and NADH at 30 °C. Nevertheless, k_cat_ and the catalytic efficiency are significantly higher for NADPH (Table 3).

#### 2.3.4. Substrate Specificity

**Figure 5:**
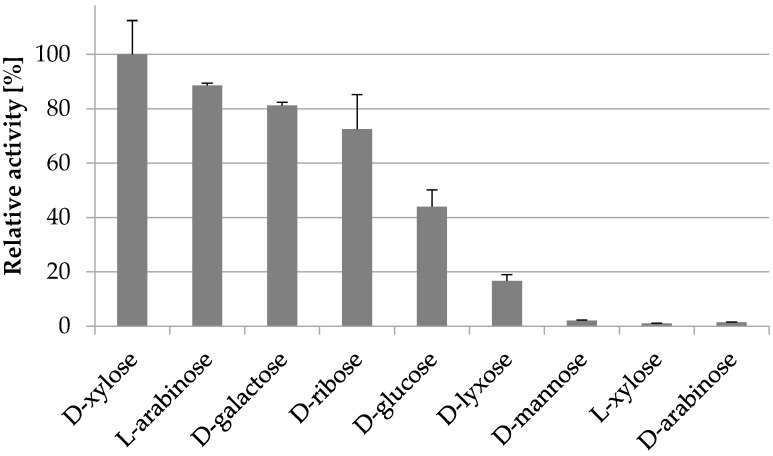
Relative activity of *Ct*XR at 0.5 M substrate concentration. 0.4 mM NADPH was used as cofactor in a 50 mM bis-tris buffer, pH 6.5, at 30 °C.

Relative activity compared to d-xylose was determined for all d-pentoses, l-xylose, l-arabinose, as well as for d-glucose, d-galactose and d-mannose (Figure 5). The enzyme shows promiscuity at high substrate concentrations, readily converting some pentoses and hexoses (l-arabinose, d-ribose, d-galactose, d-glucose), while others are converted slightly or to no extent (d-lyxose, d-mannose, l -xylose, d-arabinose).

**Figure 6:**
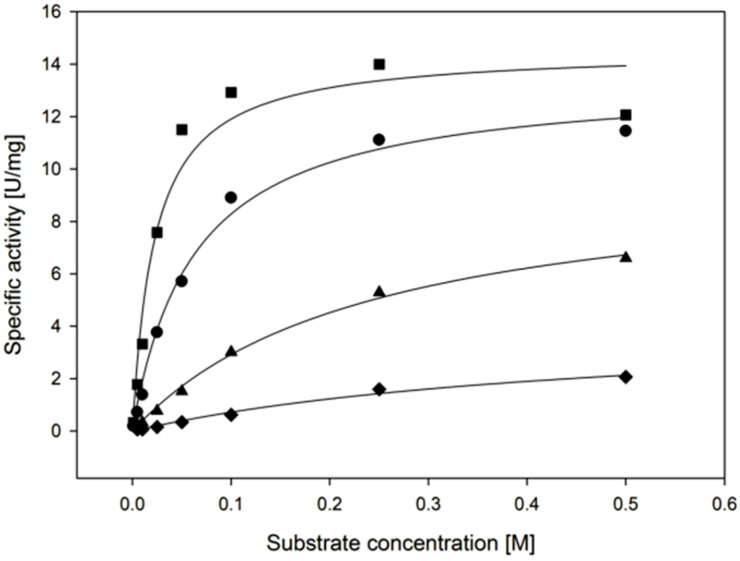
Michaelis-Menten curves with the substrates ◆ d-glucose, ▲ d-galactose, ● l-arabinose and ■ d-xylose. The concentration of sugars was varied and concentration of NADPH kept at 0.4 mM, measurements were taken at 30 °C.

To further investigate the XR´s apparent affinity towards substrates of industrial relevance, we determined K_M_, k_cat_ and catalytic efficiency for d-xylose, l-arabinose, d-galactose and d-glucose (Figure 6). These sugars represent major constituents of hemicellulose and are converted with reasonably high relative activity. A clear difference between the conversion of the pentose l-arabinose and the hexoses d-glucose and d-galactose can be seen. l-arabinose, being structurally similar to d-xylose, has a k_cat_/K_M_ value of still 33% of the value for d-xylose, whereas d-galactose and d-glucose result in only 6.3% and 1.3% of the k_cat_/K_M_ for d-xylose. For the observed cases the difference in the catalytic efficiency is mainly a result from different K_M_ values rather than differences in k_cat_, as shown in Table 4. This indicates that the reduction of the carbonyl carbon is influenced to a lesser degree, compared to the binding of the substrate itself.

#### 2.3.5. Temperature Stability

**Figure 7:**
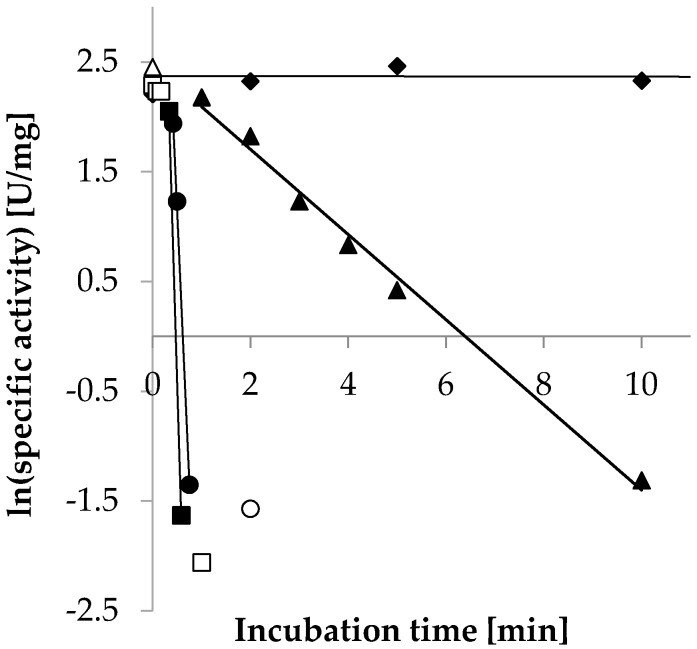
Plot of residual activity against incubation time; the decay rate λ was determined as the slope of a linear fit during the degradation phase (see Table 5). ◆ 30 °C, ▲ 55 °C, ● 65 °C and ■ 75 °C incubation temperature. Data points with empty symbols were not taken into account for the calculation of the decay rate.

The half-life (T_1/2_) of *Ct*XR was determined at 30, 55, 65 and 75 °C. Enzyme inactivation was assumed to follow first order kinetics. After plotting the natural logarithm of the specific activity against the incubation time, the decay rate λ was determined as the slope of a linear fit during the degradation phase (Figure 7). When incubated at 30 °C no loss in activity could be observed over the course of 3 h incubation time. However, stability dropped significantly when *Ct*XR was incubated at 55 °C, resulting in a half-life of 1.8 min, which decreased even further to 4.2 and 2.8 s at 65 and 75 °C, respectively (Table 5). Due to the low number of sample points caused by the fast decrease of activity, the latter two values should be considered as estimations. The comparatively low temperature stability of the enzyme is surprising, since its original host *C. thermophilum* is among the most thermophile Eukaryotes with an optimal growth temperature of 50–55 °C [1]; also a xylanase derived from *C. thermophilum* has been shown to be stable at 60 °C [26]. Possible explanations for this phenomenon could be that the pathway of xylose degradation in *C. thermophilum* is only active at temperatures significantly below the optimal growth temperature, or that the enzyme is greatly stabilized in the native environment of the host´s cytosol, making the in vitro results not transferable to natural conditions. Nevertheless, a stabilizing effect due to presence of cofactor (NADPH) or substrate (d-xylose) could not be confirmed in vitro (data not shown).

### 2.4. Structural Analysis

The initial homology model generated by SWISS-MODEL [27] with *Ca*XR as template (PDB code 1SM9, [28]; 52.7% sequence identity to *Ct*XR) provides a reliable prediction of the *Ct*XR with a global model quality estimation (GMQE) score of 0.80, and QMEAN Z-score (reliability) of −0.73.

**Figure 8:**
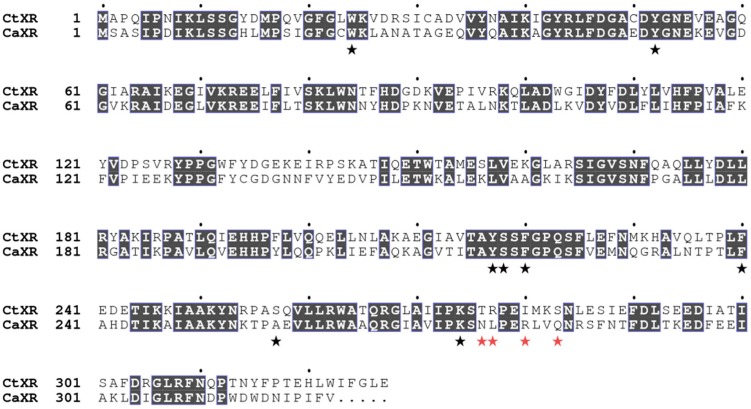
Sequence alignment for *Ct*XR and *Ca*XR (UniProt O74237). Shaded boxes represent identities. Black asterisks are residues highlighted in Figure 9 and red asterisks represent notable amino-acid replacements in the loop involved in binding the adenosine moiety of the cofactor.

The modeled nicotinamide, ribose and diphosphate regions of the cofactor-binding site in *Ct*XR are identical in sequence to that of *Ca*XR (PDB code 1MI3). Structural discrepancies are confined to the adenosine-binding site (adenine ring and ribose moieties) where amino-acid replacements in the loop comprising residues 276–283 (same numbering in *Ct*XR and *Ca*XR) are predicted to affect the precise details of adenosine binding, redox properties, and the relative preference for NAD(H) versus NADP(H). Notable *Ca*XR-to-*Ct*XR replacements include N276T, L277R, R280I and Q283S (Figure 8).

**Figure 9:**
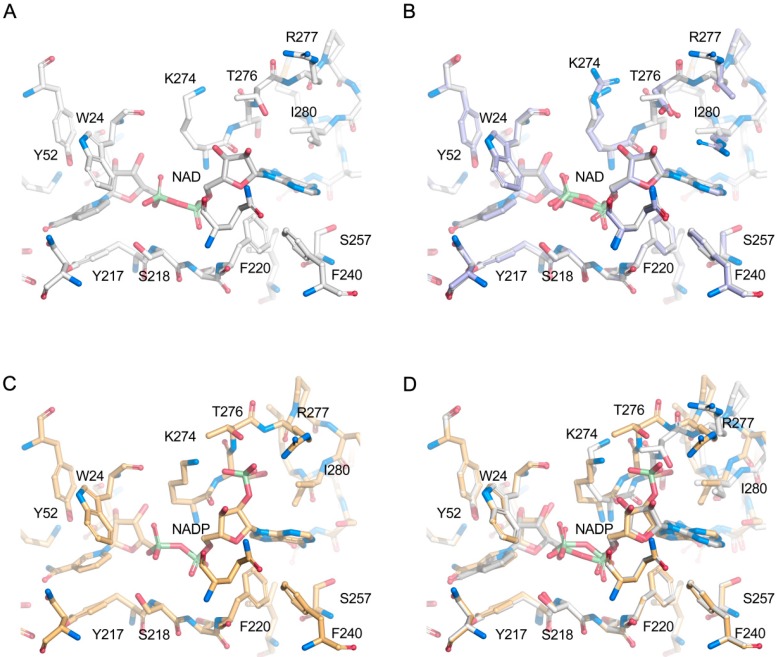
Representation of the modeled cofactor-binding site in *Ct*XR. (**A**) Details of the *Ct*XR-NAD homology model (gray). (**B**) Overlay of the *Ct*XR homology model (gray) and the *Ca*XR crystal structure (light blue). (**C**) Details of the *Ct*XR-NADP homology model (orange). (**D**) Overlay of the *Ct*XR-NAD (gray) and *Ct*XR-NADP (orange) homology models. All side-chain notations are according to the *Ct*XR sequence.

Comparing the modeled NAD(H) complex of *Ct*XR (Figure 9A) with the corresponding crystal complex of *Ca*XR (Figure 9B) indicates that the shorter threonine side chain at position 276 would be unable to provide stabilization of the adenosine ribose O2B atom to the same extent as Asn276 in *Ca*XR. Furthermore, the cation-π-stacking interaction of the Arg280 guanido group with the pyrimidine ring of the adenine-purine system in *Ca*XR is abolished by the isoleucine side chain in *CtXR*. The effects on NAD(H) binding due to the replacements of Leu277 and Gln280 by Arg and Ser in *Ct*XR, respectively, are less obvious from the homology model.

The model of *Ct*XR with bound NADP(H) was based on details of NADP(H) binding in the crystal structure of pig aldehyde reductase *Ss*ADH (PDB code 1HQT). While *Ct*XR and *Ss*ADH display more pronounced overall differences at the sequence level, the cofactor-binding site appears sufficiently similar to justify using *Ss*ADH as template for prediction of NADP(H) binding in *Ct*XR. In *Ct*XR, the ribose 2′-phosphate group of NADP(H) would receive stabilization through the donation of a hydrogen bond from Thr276 Og1 and ionic interactions with the guanido group of Arg277, and possibly Lys274 depending on its precise side-chain conformation (Figure 9C). The overall number of interactions provided for NAD(H) and NADP(H) would appear to favor NADP(H) over NAD(H) binding (Figure 9D). However, we did not observe this preference in our biochemical data (Table 2).

### 2.5. Comparison with Xylose Reductases in the Literature

Comparison with other XRs in the literature shows that k_cat_ and also k_cat_/K_M, (Xylose)_ of *Ct*XR are about average, whereas K_M, (Xylose)_ is the lowest of the reported XRs (Table 6). The pH optimum of 6.0 to 7.0 is similar to other XRs (generally between pH 5.5–6.5). Contrary to what could be expected from the thermophilicity of its natural host, the temperature stability of *Ct*XR is comparatively low and more thermostable XRs have been described (e.g., from *N. crassa* [24] or *C. tropicalis* [29]). Like many other XRs, the enzyme shows a preference for NADPH, nevertheless dual cofactor specificity can be observed: k_cat_/K_M_ is approximately 3.2-times higher when NADPH is used as cofactor instead of NADH. A ratio of 13.6 can be found for *N. crassa* [24], while an XR from *C. tenuis* shows a ratio of 1.4 [4,6] or 1.8 [28] and an XR from *Candida parapsilosis* was even reported to strongly favor NADH over NADPH as cofactor with a k_cat_/K_M, (Xylose)_ ratio of 0.013 [8].

## 4. Materials and Methods

### 4.1. Materials and Reagents

NADH, as disodium salt hydrate, L-xylose and Bradford reagent were ordered from Sigma-Aldrich (St. Louis, MO, USA); d-lyxose from Carbosynth (San Diego, CA, USA); D-arabinose from TCI Europe (Antwerp, Belgium); NADPH, as tetrasodium salt, and all other chemicals were ordered from Carl Roth (Karlsruhe, Germany).

### 4.2. Construction of the Expression Plasmid

The XR gene was identified in the genome of *C. thermophilum* DSM 1495 by homology search and the construct was ordered from genscript (Piscataway, NJ, USA), codon optimized for *Escherichia coli* (wild type and codon optimized nucleotide sequences as well as the amino acid sequence of *Ct*XR can be found as Supplementary Materials). The open reading frame was amplified and restriction sites. NcoI and XhoI were added by polymerase chain reaction on the 5′ and 3′ end, respectively. For the construction of the expression plasmid, the amplicon was integrated in a pET28a vector containing a C-terminal hexahistidin (His6) tag. The expression plasmid was transformed into chemically competent cells *E. coli* BL21(DE3) using a heat shock protocol: 2 µL of plasmid (160 ng/µL) were added to 50 µL cell suspension. After 30 min incubation on ice, heat shock was performed at 42 °C for 45 s. Cells were transferred back on ice for 2 min and subsequently diluted with 950 µL SOC medium. After 1 h at 37 °C cells were plated on LB-agar plates containing 100 mg/L ampicillin for picking transformants after overnight incubation at 37 °C (10, 50 and 200 µL per plate). Correct insertion and integration were verified by sequencing (Microsynth, Balgach, Switzerland).

### 4.3. Protein Production

The protein was produced via fed-batch fermentation in a 15 L Sartorius Cplus bioreactor with 8 L working volume (Satorius, Göttingen, Germany). The preculture was grown in a shake flask for 20 h at 37 °C and 230 rpm in 500 mL of a medium according to DeLisa et al. [31] containing 8 g/L glucose and 100 mg/L ampicillin, adjusted to pH 7.2. The preculture was transferred aseptically to the culture vessel yielding a total starting volume for the batch phase of 5 L, containing 20 g/L glucose and 100 mg/L ampicillin. Batch phase was performed at 37 °C, followed by an uninduced fed-batch phase of 14 hours (exponential feed, µ = 0.1 h^−1^) to increase cell density to 35 g/L DCW and finally an induced fed-batch phase (exponential feed, µ = 0.04 h^−1^), performed at 25 °C to increase protein solubility. Production of the recombinant protein was induced by aseptic addition of 0.1 mM IPTG (final concentration).

Cells were harvested by centrifugation (4500× *g*, 4 °C, 30 min) 14 h after induction at a final cell density of approximately 62 g/L DCW. The cell pellet was stored at −20 °C for later use.

Dissolved oxygen (dO_2_) was measured with a dissolved oxygen electrode Visiferm DO425 (Hamilton, Reno, NV, USA) and dO_2_ levels were maintained above 30% by aerating with 7.5 L/min pressurized air. Air was substituted with pure oxygen if necessary. pH was monitored with an Easyferm electrode (Hamilton) and kept at 7.2 by addition of NH_4_OH (12.5% *v*/*v*) via the pump module of the bioreactor. Feed was supplied via a Preciflow peristaltic pump (Lambda, Switzerland) following a feed-forward controlled exponential feeding strategy. Mixing was performed at 1400 rpm. CO_2_ and O_2_ content in the offgas were analyzed with a DASGIP GA, (Eppendorf, Hamburg, Germany). Process parameters were adjusted and recorded via the process information management system Lucullus (Securecell, Schlieren, Switzerland).

### 4.4. Protein Purification

230 g frozen biomass were thawed and resuspended in a loading buffer (50 mM bis-tris, 30 mM imidazole, 5% *w*/*v* glycerol, pH 6.5) to a total volume of 500 mL. Cell disruption was carried out with a homogenizer (4 passages, 1500 bar; PandaPLUS 2000, GEA Mechanical Equipment, Parma, Italy).

Cell debris was removed via tangential flow filtration (0.2 µL cutoff, max. transmembrane pressure 1.5 bar, max. flow rate 0.9 mL/min), resulting in 400 mL cell free crude extract.

For protein purification an Äkta Pure25 (GE Healthcare, Solingen, Germany) was used. IMAC was used as the first purification step, followed by size exclusion chromatography. 365 mL crude extract was loaded on a His Trap FF Crude column (5 mL column volume (CV); GE Healthcare) with a flow rate of 15 cm/h. After washing with 2 CVs with loading buffer, elution was carried out with a linear gradient over 6 CVs (0–100% elution buffer; 50 mM bis-tris, 500 mM imidazole, 5% *w*/*v* glycerol, pH 6.5). Protein concentration was monitored in the flowthrough at 280 nm and the target protein was collected manually in a single fraction of 8 mL.

After concentration to a volume of 2 mL with centrifugal filters with a 10 kDa cut off (Amicon Ultra 15, Merck, Darmstadt, Germany), the protein was purified via size exclusion chromatography (SEC). 2 mL protein solution were loaded on a high load superdex 75 pg column (120 mL CV; GE Healthcare, Germany) with a flow rate of 30 cm/h (running buffer: 25 mM tris-HCl, 150 mM NaCl, 5% *w*/*v* glycerol, pH 7.4). Fractions of 1 mL were collected.

Protein purity was assessed with SDS-PAGE. Samples of all stages of the purification process were diluted appropriately. After dilution with 2x Laemmli buffer samples were incubated at 95 °C for 5 min. 10 µL of each sample and 4 µL SeeBlue Plus 2 protein standard (Thermo Scientific, Waltham, MA, USA) were loaded onto 4–15% Mini-PROTEAN TGX precast gels (Bio-Rad, Hercules, CA, USA). Gels were run for 45 min at 160 V and stained with Coomassie Sensitive stain (50 g/L aluminum sulfate (14–18 hydrate), 100 mL/L ethanol, 23.5 mL/L orthophosphoric acid, 0.2 g/L Coomassie blue G250) over night, washed with water and analyzed with Gel Doc XR system and ImageLab software (Bio-Rad, Hercules, CA, USA).

### 4.5. Data Analysis

For evaluation of purification steps, enzyme activity (U/mL) and protein concentration (mg/mL) in crude extract, IMAC eluate, spin filter retentate and collected SEC fraction were determined and the respective specific activities (U/mg) were determined. By comparing the specific activity of the crude extract to those of the fractions the purification factor (PF) was calculated Equation (1).

(1) $$Purification\;factor\;\left(PF\right)=\frac{specific\;activity_{fraction}}{specific\;activity_{crude\;extract}}$$

To quantify the loss of target protein during the purification, the recovery of the catalytic activity was calculated according to Equation (2).

(2) $$Recovery\;\left(\%\right)=\frac{total\;activity_{fraction}}{total\;activity_{crude\;extract}}\times 100.$$

### 4.6. Protein Quantification

Protein concentration was measured photometrically (Genesys 20, Thermo Scientific) using the Bradford Coomassie Blue assay with bovine serum albumin as standard. When necessary, samples were diluted with their respective buffers.

### 4.7. Biochemical Characterization

#### 4.7.1. Enzyme Activity

Enzyme activity, proportional to the conversion of the cofactors NADPH or NADH, was determined photometrically following the decrease of absorption at 340 nm (point of max. absorption of NADPH and NADH; ε_340_ = 6.22 mM^−1^·cm^−1^) in duplicates. The activity was calculated according to Equations 3 and 4 (volumetric and specific activity, respectively). The mean specific activity was plotted against the substrate concentration (7 to 8 data pairs) and by fitting a Michaelis-Menten curve to the data points, V_max_ and K_M_ were determined using the program SigmaPlot 12.5 (Systat Software, San Jose, CA, USA). The standard errors for K_M_ and k_cat_ specified in the Results section were calculated per default, with SigmaPlot describing the quality of the fit. k_cat_ and the catalytic efficiency (k_cat_/K_M_) were calculated according to Equations 5 and 6. For the calculation of the confidence interval for k_cat_/K_M_ both the errors from k_cat_ and K_M_ were considered using the formula for the propagation of uncertainty, as shown in Equation (7).

(3) $$\mathrm{volumetric}\;\mathrm{activity}=\frac{\Delta \mathrm{Abs}\cdot V_{\mathrm{total}}}{\varepsilon \cdot V_{\mathrm{enzyme}}\cdot d}$$

(4) $$\mathrm{specific}\;\mathrm{activity}=\frac{\mathrm{volumetric}\;\mathrm{activity}}{c_{\mathrm{enzyme}}}$$

(5) $$k_{\mathrm{cat}}=V_{\max}\cdot M_{\mathrm{enzyme}}$$

(6) $$\mathrm{specific}\;\mathrm{activity}=\frac{\mathrm{volumetric}\;\mathrm{activity}}{c_{\mathrm{enzyme}}}$$

(7) $$\sigma_{\mathrm{catalytic}\;\mathrm{efficiency}}=\sqrt{{\left(\frac{1}{K_{M}}\right)}^{2}\cdot S{E_{k_{\mathrm{cat}}}}^{2}+{\left(-\frac{k_{\mathrm{cat}}}{{K_{M}}^{2}}\right)}^{2}\cdot S{E_{K_{M}}}^{2}}$$

ΔAbs: Change in absorption (min^−1^); V_total_: Assay volume (mL); ε: Extinction coefficient of NAD(P)H at 340 nm (6.22 L·mmol_NAD(P)H_^−1^·cm^−1^); V_enzyme_: Volume of enzyme solution (mL_XR_); d: Path length (1 cm); Volumetric activity: Activity per mL of enzyme solution (µmol_NAD(P)H_·min^−1^·mL_XR_^−1^ = U/mL); c_enzyme_: Concentration of enzyme solution (mg_XR_/mL_XR_); Specific activity: Activity per mg of enzyme (µmol_NAD(P)H_·min^−1^·mg_XR_^−1^ = U/mg); V_max_: Maximum enzyme activity (mol_NAD(P)H_·s^−1^·mg_XR_^−1^); M_enzyme_: Molecular mass of enzyme (39,244 Da); k_cat_: Turnover number (mol_NAD(P)H_·mol_XR_^−1^·s^−1^); K_M_: Apparent affinity constant (M_substrate_); k_cat_/K_M_: Catalytic efficiency (mol_NAD(P)H_·mol_XR_^−1^·M_substrate_^−1^·s^−1^); σ_catalytic efficiency_: confidence interval of k_cat_/K_M_ (mol_NAD(P)H_·mol_XR_^−1^·M_substrate_^−1^·s^−1^); SE: standard error (of k_cat_ (mol_NAD(P)H_·mol_XR_^−1^·s^−1^) or K_M_ (M_substrate_)).

The purified enzyme was incubated in 50 mM bis-tris buffer pH 6.5 containing different amounts of cofactor and d-xylose or alternative substrates and analyzed in a thermoregulated photometer (V-630 Spectrophotometer, Jasco, Pfungstadt, Germany). Depending on the investigated cofactor and substrate the enzyme concentration in the assay ranged from 0.564 to 45.2 µg/mL. One unit of activity was defined as the amount of enzyme catalyzing the oxidation of 1 μmol NAD(P)H/min. The kinetic parameters K_M_, k_cat_ and catalytic efficiency were determined at 30 and at 55 °C (the highest temperature at which a characterization was justified due to the enzyme´s moderate thermostability). d-Xylose (0.001–0.5 M) was used as substrate and NADPH as cofactor (0.4 mM). To ensure that the change in absorption was not caused by thermal degradation of NAD(P)H, activity assays containing no *Ct*XR were performed as blanks. In accordance with the literature [32], thermal cofactor degradation was insignificant under assay conditions compared to enzymatic activity.

Activity of samples derived during protein purification was measured at 30 °C in 50 mM bis-tris buffer pH 6.5 containing 0.5 M d-xylose and 0.4 mM NADPH.

#### 4.7.2. pH Optimum

pH range was determined by measuring the enzymatic activity at 30 °C in a citric acid-disodium phosphate buffer (McIlvaine buffer) [33] (pH 2.4–8.1) and 50 mM glycine buffer (pH 8.5–10.0). NADPH (0.4 mM) was used as cofactor and d-xylose (0.5 M) as substrate. Measurements were performed in triplicates. Additionally, the pH optimum was determined in 50 mM citrate and phosphate buffers.

#### 4.7.3. Cofactor Preference

Kinetic parameters K_M_, k_cat_ and catalytic efficiency were determined for the cofactors NADPH (0.01–0.6 mM) and NADH (0.01–0.8 mM) in a 50 mM bis-tris buffer, pH 6.5, at 65 °C.

#### 4.7.4. Substrate Specificity

We determined relative activities for the substrates d-xylose, l-xylose, d-arabinose, l-arabinose, d-ribose, d-lyxose, d-glucose, d-galactose and d-mannose. Measurements for the determination of the relative activity were performed in triplicates using 0.5 M substrate concentration and 0.4 mM NADPH as cofactor in a 50 mM bis-tris buffer, pH 6.5, at 30 °C. Additionally, kinetic parameters K_M_, k_cat_ and catalytic efficiency were determined for the substrates d-xylose, l-arabinose, d-galactose and d-glucose (each 0.001–0.5 M) using NADPH as cofactor (0.4 mM).

#### 4.7.5. Temperature Stability

The enzyme solution (0.2825 mg/mL; without substrate or cofactor) was incubated at 30, 55, 65 and 75 °C. At different time points aliquots were drawn and the catalytic activity was immediately determined at 30 °C. NADPH (0.4 mM) was used as cofactor and d-xylose (0.5 M) as substrate. For the calculation of the temperature stability of the enzyme, data acquired during the first ~15 s of incubation were omitted, since this was the time required to fully heat the test tubes (including 0.5 mL sample volume) on the thermoblock. The half-life (T_1/2_) of *Ct*XR was determined at 30, 55, 65 and 75 °C according to Equation (8).

(8) $$T_{1/2}=-\frac{\ln\left(2\right)}{\lambda }$$

λ: Decay rate (min^−1^); T_1/2_: Half-life (min)

### 4.8. Homology Modeling

An initial homology model of *Ct*XR was produced using the SWISS-MODEL server (https://swissmodel.expasy.org) [26] that was further optimized manually using *COOT* (version 0.8.9.1) [34]. Based on the optimized model, homology models of *Ct*XR in complex with either NAD or NADP were generated manually by careful inspection of NAD binding in xylose reductase from *Candida tenuis* (*Ca*XR; PDB code 1MI3, [35]; 1SM9, [28]) and NADP binding in aldehyde reductase from *Sus scrofa* (*Ss*ADH; PDB code 1HQT; [35]).

## 5. Conclusions

The particular xylose reductase from *C. thermophilum* was chosen for this study, since we were looking for a thermostable enzyme for the conversion of d-xylose to xylitol for the utilization in high temperature processes operating at around 75 °C. The enzyme exhibits dual cofactor specificity for NADPH (K_M_ = 35.8 ± 7.6 mM) and NADH (K_M_ = 121 ± 12 mM), has reduced activity on l-arabinose and residual activity on d-galactose and d-glucose, and optimal activity between pH 6.0 and 7.0. Unfortunately, with a half-life of only several seconds at 75 °C, the investigated *Ct*XR cannot be used in the desired high temperature environments. Under thermophilic conditions XRs from *N. crassa* and *C. tropicalis*, described by Woodyer et al. [24] and Zhang et a. [29], respectively, have similar or even higher k_cat_/K_M_ values compared to *Ct*XR and can potentially be deployed in more thermophilic processes due to higher stability.

## Figures and Tables

**Table 1:** Summary of the purification process of *Ct*XR. Recovery and purification factor are given relative to the activity in the crude extract and, in brackets, for retained fractions relative to the preceding fraction.

	Total Protein	Specific Activity	Total Activity	Recovery	Purification Factor
	(mg)	(U/mg)	(U)	(%)	( )
Crude extract	4296	0.08	359	100	1
IMAC Flow through	4158	0.09	392	109	1.1
IMAC Eluate 1 (pre elution peak)	11.85	0.28	3.3	0.9	3.4
IMAC Eluate 2 (post elution peak)	3.96	0.12	0.5	0.1	1.4
IMAC Elution peak	55.7	1.49	83.1	23.1 (23.1)	17.8 (17.8)
Spin filter Flow through	25.1	2.12	53.1	14.8	25.3
Spin filter retentate	34.1	0.76	26.0	7.2 (31.3)	9.1 (0.5)
SEC Pool 1 (discarded fraction)	13.4	0.76	10.1	2.8	9.0
SEC Pool 2 (discarded fraction)	5.20	0.86	4.5	1.2	10.3
SEC Peak (collected fraction)	4.51	2.13	9.6	2.7 (36.9)	25.5 (2.8)

**Table 2:** Kinetic parameters of the xylose reductase from *C. thermophilum*. d-Xylose (varied between 0.001–0.5 M).

Temperature	k_cat_ (s^−1^)	K_M, (Xylose)_ (mM)	k_cat_/K_M, (Xylose)_ (mM^−1^·min^−1^)
30 °C	11.4 ± 1.1	22.3 ± 6.1	31 ± 9
55 °C	35.2 ± 1.4	25.4 ± 4.0	83 ± 14

**Table 3:** Cofactor preference of the investigated xylose reductase. Measurements were taken at 30 °C.

Cofactor	k_cat_ (s^−1^)	K_M, (Cofactor)_ (µM)	k_cat_/K_M, (Cofactor)_ (µM^−1^·min^−1^)
NADPH	11.4 ± 1.1	135 ± 44	5.1 ± 1.7
NADH	3.2 ± 0.1	119 ± 6	1.6 ± 0.1

**Table 4:** Substrate specificity of the investigated xylose reductase. The enzyme showed a clear preference for d-xylose (k_cat_/K_M_). Measurements were taken at 30 °C.

Substrate	k_cat_ (s^−1^)	K_M, (Substrate)_ (mM)	k_cat_/K_M, (Substrate)_ (mM^−1^·min^−1^)
d-xylose	9.2 ± 0.6	22.3 ± 6.1	26 ± 7
l-arabinose	8.8 ± 0.4	62.5 ± 7.7	8.4 ± 1.1
d-galactose	6.5 ± 0.4	241 ± 29	1.6 ± 0.2
d-glucose	2.7 ± 0.5	471 ± 141	0.3 ± 0.1

**Table 5:** Decay rate λ and half-life T_1/2_ at 30 °C, 55 °C, 65 °C and 75 °C.

Temperature	Decay Rate (λ) (s^−1^)	Half-Life (T_1/2_)
◆ 30 °C	n.a.	n.a.
▲ 55 °C	−6.5 × 10^−3^	1.8 min
● 65 °C	−0.17	4.2 s
■ 75 °C	−0.25	2.8 s

n.a. not applicable (no loss of activity over 3 h).

**Table 6:** Comparison of xylose reductases from various hosts: If possible, missing data were complemented with calculation based on the accessible values. In case of publication of multiple values for identical kinetic parameters, the mean value is shown in the table.

Organism and Source	Cofactor	k_cat_ (s^−1^)	K_M, (Xylose)_ (mM)	k_cat_/K_M, (Xylose)_ (mM^−1^·min^−1^)	K_M, (Cofactor)_ (µM)	k_cat_/K_M, (Cofactor)_ (µM^−1^·min^−1^)	Assay Conditions		Optimum		Temp. Stability
							Temp. (°C)	pH	Temp. (°C)	pH	
*Chaetomium thermophilum* (this work)	NADPH	35.2	25.4	83			55	6.5	55	6.5	No loss of activity after 3 h at 30 °C T_1/2_ = 1.8 min (55 °C)
	NADH	3.2			119	1.6	30				
*Neurospora crassa* [24]	NADPH	60	34	106	1.8	2000	25	6.3	45–55	5.5	T_1/2_ = 49 min (40 °C)
	NADH	5.2	37	8.4	16	19.5					
*Candida tenuis* [4,6]	NADPH	21.7	72	18.1	4.8	271	25	7.0	50	6.0	Start of decrease at 30-35 °C
	NADH	18.2	87	12.6	25.4	43					
*Candida tropicalis* [30]	NADPH	49	30	99	18	329	RT	7.0	-	6.0	Complete loss after 1 h at 60 °C
*Candida intermedia* [5]	NADH	16.5	30	31.8	10	101	25	7.0	-	-	-
*Pichia stipitis* [7]	NADPH	25.2	42	36	9	167	30	6.0	-	6.0	-
*Candida parapsilosis* [8]	NADPH	4.6	244	1.14	36.5	7.6	37	6.0	-	6.0	T_1/2_ = 4.5 h (45 °C); 2 min (50 °C)
	NADH	49	32	87.6	3.3	939					
*Candida tenuis* [28]	NADPH	13	96	8.1	3	260	25	7.0	-	-	-
	NADH	11	142	4.6	38	17					
*Candida tropicalis* [29]	NADPH	240	31.5	457	45.5	316	45	5.5	45	5.5	T_1/2_ = 15 min (60 °C)
	NADH	-	-	-	162	-					

RT room temperature; - not determined.

## Supplementary Materials

Supplementary materials can be found at http://www.mdpi.com/1422-0067/20/1/185/s1.

## Abbreviations

XR	xylose reductase
His6	hexahistidine
NADPH	nicotinamide adenine dinucleotide phosphate (reduced)
NADH	nicotinamide adenine dinucleotide (reduced)
PDB	Protein Data Bank
EC	Enzyme Commission (number)
NAD^+^	nicotinamide adenine dinucleotide (oxidized)
*Ct*XR	xylose reductase from *Chaetomium thermophilum*
DCW	dry cell weight (g/L)
SDS-PAGE	sodium dodecyl sulfate-polyacrylamide gel electrophoresis
IMAC	immobilized metal affinity chromatography
SEC	size exclusion chromatography
V_max_	maximum enzyme activity (mol_NAD(P)H_·s^−1^·mg_XR_^−1^)
k_cat_	turnover number (mol_NAD(P)H_·mol_XR_^−1^·s^−1^)
K_M_	substrate affinity (M_substrate_)
k_cat_/K_M_	catalytic efficiency (mol_NAD(P)H_·mol_XR_^−1^·M_Substrate._^−1·s−1^)
SE	standard error
*Ca*XR	xylose reductase from *Candida tenuis*
GMQE	global model quality estimation
*Ss*ADH	aldehyde reductase from *Sus scrofa*
CV	column volume (mL)
NAD(H)	nicotinamide adenine dinucleotide
NADP(H)	nicotinamide adenine dinucleotide phosphate
