# Supplementary material for: Kinetics and Predicted Structure of a Novel Xylose Reductase from Chaetomium thermophilum
Source: Int J Mol Sci. 2019 Jan 6;20(1):185. doi: 10.3390/ijms20010185 (PMC6337131; doi:10.3390/ijms20010185)
Supplement: Supplementary file 1 [file ijms-20-00185-s001.pdf]

1) Nucleotide sequence of the putative xylose reductase gene from *Chaetomium thermophilum* DSM 1495 (GenBank accession number XM_006695955.1):

ATGGCTCCCCAGATCCCCAACATTAAGCTCAGCAGCGGCTATGACATGCCCC
AGGTGGGCTTTGGACTGTGGAAGGTCGACCGCTCGATCTGCGCCGACGTCGT
CTACAACGCAATTAAGATTGGCTACCGCCTGTTTGACGGCGCTTGCGACTATG
GCAACGAAGTCGAGGCCGGCCAGGGCATTGCCCGCGCCATCAAGGAGGGCA
TCGTGAAGCGTGAGGAGCTCTTCATCGTGTCCAAGCTCTGGAACACCTTCCAC
GACGGCGACAAGGTCGAGCCCATCGTCCGCAAGCAGCTCGCCGACTGGGGC
ATTGACTACTTCGACCTCTACCTCGTCCACTTCCCTGTCGCCCTCGAGTACGTC
GACCCCTCGGTCCGCTACCCGCCCGGCTGGTTCTACGATGGCGAGAAGGAGA
TCCGCCCCAGCAAGGCCACCATCCAGGAGACCTGGACCGCCATGGAGTCGC
TCGTTGAGAAGGGTCTGGCCCGCAGCATTGGTGTCTCCAACTTCCAGGCCCA
GCTCCTCTACGACCTGCTGCGCTACGCCAAGATCCGCCCGGCCACCCTTCAG
ATCGAGCATCACCCCTTCCTCGTCCAGCAGGAGCTGCTCAACCTGGCCAAGG
CCGAGGGCATTGCCGTGACTGCCTACAGCTCATTCGGCCCTCAGAGCTTCCTC
GAGTTCAACATGAAGCACGCCGTGCAGCTCACCCCGCTCTTCGAGGACGAGA
CCATCAAGAAGATCGCCGCCAAGTACAACCGTCCTGCTTCGCAGGTTCTCCT
GCGCTGGGCCACTCAGCGCGGTCTGGCCATTATCCCCAAGAGCACGCGCCCC
GAGATCATGAAGTCCAACCTCGAGAGCATCGAGTTCGACCTCAGCGAGGAA
GATATTGCCACCATCTCGGCCTTCGACCGCGGCCTGCGCTTCAACCAGCCCA
CAAACTACTTCCCCACCGAGCACCTCTGGATCTTTGGCTAA

2) Nucleotide sequence of the expressed xylose reductase gene from *Chaetomium thermophilum* (after codon optimization):

ATGGCTCCTCAAATACCTAATATTAAGTTATCAAGTGGTTATGATATGCCTCA
AGTTGGTTTTGGTTTATGGAAAGTAGATAGAAGTATTTGTGCTGATGTAGTTT
ATAATGCAATTAAGATAGGATATAGATTATTTGATGGAGCTTGTGATTATGGT
AATGAAGTAGAAGCAGGACAAGGTATAGCTAGAGCAATAAAAGAAGGTATT
GTAAAGAGAGAAGAATTATTTATTGTTTCTAAGTTATGGAATACTTTTCATGA
TGGAGATAAGGTAGAACCAATTGTTAGAAAGCAATTAGCTGATTGGGGTATT
GATTATTTTGATTTATATTTAGTACATTTTCCAGTTGCATTAGAATATGTAGAT
CCTTCAGTTAGATATCCACCTGGATGGTTTTATGATGGTGAAAAGGAAATAA
GACCTAGTAAGGCTACAATACAAGAAACTTGGACAGCAATGGAATCTTTAGT
AGAAAAGGGATTAGCTAGAAGTATAGGTGTTTCTAATTTTCAAGCACAATTA
TTATATGATTTATTAAGATATGCTAAGATAAGACCAGCAACTTTACAAATAG
AACATCATCCATTTTTAGTACAACAAGAATTATTAAATTTAGCTAAAGCAGA
AGGAATAGCTGTTACAGCATATTCTTCATTTGGTCCACAATCATTTTTAGAAT
TTAATATGAAGCATGCTGTACAATTAACTCCTTTATTTGAAGATGAAACAATA
AAGAAAATAGCTGCAAAGTATAATAGACCAGCTTCACAAGTTTTATTAAGAT
GGGCTACTCAAAGAGGATTAGCAATAATTCCAAAGAGTACAAGACCTGAAA
TAATGAAGTCAAATTTAGAAAGTATAGAATTTGATTTATCTGAAGAAGATAT
TGCTACTATATCAGCATTTGATAGAGGTTTAAGATTTAATCAACCTACTAATT

ATTTTCCTACAGAACATTTATGGATATTTGGACTCGAGCACCACCACCACCAC CACTGA

3) Amino acid sequence of the expressed xylose reductase gene from *Chaetomium thermophilum*:

Met A P Q I P N I K L S S G Y D Met P Q V G F G L W K V D R S I C A D V V Y N A I K I G Y R L F D G A C D Y G N E V E A G Q G I A R A I K E G I V K R E E L F I V S K L W N T F H D G D K V E P I V R K Q L A D W G I D Y F D L Y L V H F P V A L E Y V D P S V R Y P P G W F Y D G E K E I R P S K A T I Q E T W T A Met E S L V E K G L A R S I G V S N F Q A Q L L Y D L L R Y A K I R P A T L Q I E H H P F L V Q Q E L L N L A K A E G I A V T A Y S S F G P Q S F L E F N Met K H A V Q L T P L F E D E T I K K I A A K Y N R P A S Q V L L R W A T Q R G L A I I P K S T R P E I Met K S N L E S I E F D L S E E D I A T I S A F D R G L R F N Q P T N Y F P T E H L W I F G L E H H H H H H Stop

4) pH optimum in alternative buffer systems

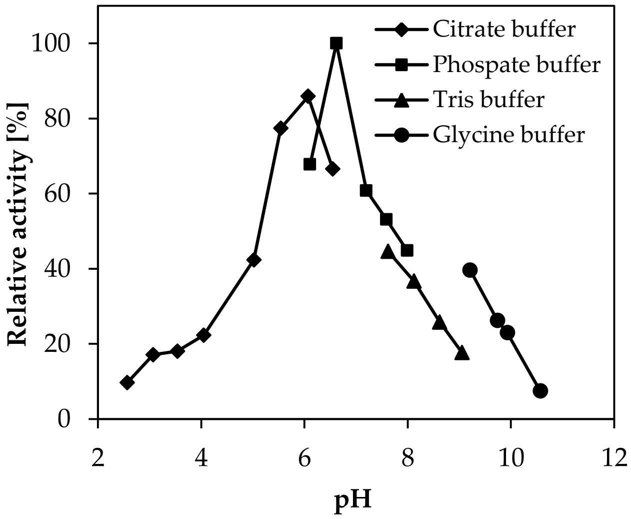

Figure S1: Xylose reductase activity at different pH values in alternative buffer systems. Measurements were taken at 30 °C in a set of different 50 mM buffers covering a pH range from 2.5 to 10.5 in steps of 0.5 pH units: ◆ citrate (2.5 – 6.5), ■ phosphate (6.0 – 8.0), ▲ tris (7.5 – 9.0) and ● glycine (9.0 – 10.5). NADPH was used as cofactor and D-xylose as substrate.
